# Peer review reduces spin in PCORI research reports

**DOI:** 10.1186/s41073-021-00119-1

**Published:** 2021-12-01

**Authors:** Evan Mayo-Wilson, Meredith L. Phillips, Avonne E. Connor, Kelly J. Vander Ley, Kevin Naaman, Mark Helfand

**Affiliations:** 1 Department of Epidemiology and Biostatistics, Indiana University School of Public Health-Bloomington, 1025 E. 7th Street, PH 179D, Bloomington, Indiana 47405 USA; 2 Department of Epidemiology, Johns Hopkins Bloomberg School of Public Health, Baltimore, MD USA; 3 Department of Medical and Clinical Informatics and Clinical Epidemiology, Oregon Health & Science University School of Medicine, Portland, Oregon USA; 4 Department of Counseling and Educational Psychology, Indiana University School of Education-Bloomington, Bloomington, Indiana USA; 5 Oregon Health & Science University School of Medicine, Portland, Oregon USA

**Keywords:** Peer review, Spin, Reporting bias, Research funding, Patient centered outcomes research institute (PCORI), Interventions

## Abstract

**Background:**

The Patient-Centered Outcomes Research Institute (PCORI) is obligated to peer review and to post publicly “Final Research Reports” of all funded projects. PCORI peer review emphasizes adherence to PCORI’s Methodology Standards and principles of ethical scientific communication. During the peer review process, reviewers and editors seek to ensure that results are presented objectively and interpreted appropriately, e.g., free of spin.

**Methods:**

Two independent raters assessed PCORI peer review feedback sent to authors. We calculated the proportion of reports in which spin was identified during peer review, and the types of spin identified. We included reports submitted by April 2018 with at least one associated journal article. The same raters then assessed whether authors addressed reviewers’ comments about spin. The raters also assessed whether spin identified during PCORI peer review was present in related journal articles.

**Results:**

We included 64 PCORI-funded projects. Peer reviewers or editors identified spin in 55/64 (86%) submitted research reports. Types of spin included reporting bias (46/55; 84%), inappropriate interpretation (40/55; 73%), inappropriate extrapolation of results (15/55; 27%), and inappropriate attribution of causality (5/55; 9%). Authors addressed comments about spin related to 47/55 (85%) of the reports.

Of 110 associated journal articles, PCORI comments about spin were potentially applicable to 44/110 (40%) articles, of which 27/44 (61%) contained the same spin that was identified in the PCORI research report. The proportion of articles with spin was similar for articles accepted before and after PCORI peer review (63% vs 58%).

**Discussion:**

Just as spin is common in journal articles and press releases, we found that most reports submitted to PCORI included spin. While most spin was mitigated during the funder’s peer review process, we found no evidence that review of PCORI reports influenced spin in journal articles. Funders could explore interventions aimed at reducing spin in published articles of studies they support.

**Supplementary Information:**

The online version contains supplementary material available at 10.1186/s41073-021-00119-1.

## Background

The Patient-Centered Outcomes Research Institute (PCORI) was authorized by the U.S. Congress in 2010. Since then, PCORI has provided over $2.8 billion in funding for research related projects [1]. A 10-year re-authorization sustains PCORI’s role as a leading supporter of stakeholder-driven investigator-initiated health research.

Following its legal mandate, PCORI is obligated to peer review and to post publicly “Final Research Reports” that enable readers to understand the activities, methods, results, and conclusions of funded projects without consulting other resources [2]. Publicly posted PCORI research reports are open access and indexed with Digital Object Identifiers (DOIs). Investigators are also encouraged to disseminate their research through journal articles and other media (e.g., conference presentations and websites). These methods are especially important for disseminating research to fellow scientists because journal articles might be more widely read, and more widely cited, compared with PCORI research reports.

PCORI research reports differ from journal articles in three ways: comprehensiveness, length, and target audience. PCORI research reports must report all aims and outcomes described in the PCORI-approved research protocol. Consequently, PCORI research reports are long documents with a limit of 15,000 words for the main body of the report. By comparison, a journal article might describe part of a project (e.g., one of multiple project aims) and might be limited in length (e.g., to 3000 words). PCORI research reports are written for readers with general scientific knowledge, who may not have expertise in the specific field represented in the report. By contrast, journal articles are often written for specialists.

Although modeled after leading peer reviewed journals, PCORI peer review of Draft Final Research Reports (DFRRs) differs from traditional peer review of academic medical journal articles. Foremost, journal editors have the option to reject submitted manuscripts for methodological failings, general poor quality, or not reporting novel or important findings [3, 4]. By contrast, PCORI editors cannot reject DFRRs, all of which will eventually be published on the PCORI website regardless of methodological limitations or the overall confidence in the study findings.

PCORI peer review emphasizes adherence to PCORI’s Methodology Standards [5] and principles of scientific integrity to ensure that results are presented objectively and clearly, and that conclusions are aligned with results. Peer reviewers typically include a patient representative, one or more subject matter experts, and one or more methodologists. After the first submission of a DFRR, a PCORI associate editor reviews the report and synthesizes comments from peer reviewers about methodological and reporting issues, and the associate editor advises investigators how to address the comments and recommendations. Later, the associate editor reads a revised version of the DFRR and may request additional revisions. This process continues until the associate editor determines that the report meets minimum standards, and the DFRR is accepted by PCORI peer review. Then, the report is reviewed by the PCORI Director of Peer Review, who might request one or more additional rounds of revision before approving it for public posting (i.e., PCORI Final Approval). When a report is published, PCORI also posts a summary of the peer review findings on its website.

Peer review aims to address many potential issues, including “spin,” which has been defined elsewhere as “reporting practices that distort the interpretation of results and mislead readers so that results are viewed in a more favorable light.” [6, 7] Several types of spin are common in peer-reviewed biomedical literature [6, 8–10]. Chiu and colleagues described four types that we evaluated in our study: reporting practices that distort the interpretation of results and create misleading conclusions suggesting a favorable result; discordance between results and their interpretation with the interpretation being more favorable than the results; attribution of causality when study design does not allow for it; and overinterpretation or inappropriate extrapolation of results [7].

PCORI’s requirement to fully report results may affect spin in unpredictable ways. By definition, the requirement for public posting of PCORI research reports (i.e., including all study aims and outcomes) should prevent selective non-reporting of studies and results. On the other hand, investigators might interpret results in a way that helps justify applications for future PCORI dissemination and implementation awards, or that helps justify applications for future research awards from PCORI and other funders.

The likelihood that peer reviewers detect spin in DFRRs, and that journal articles emanating from PCORI-funded research contain spin, is unknown. Moreover, it is unknown whether and how PCORI’s peer review process of DFRRs might affect associated journal articles.

Our main goals in this study were to assess [1] the frequency with which peer reviewers and editors identified spin during review of PCORI research reports and [2] whether investigators addressed reviewer comments related to spin in their PCORI research reports. A secondary goal was to investigate whether PCORI peer review had an effect on spin in associated journal articles.

## Methods

### Eligibility criteria

To be eligible, a PCORI-funded research project must have submitted a DFRR that completed peer review by the end of April 2018 and must have had at least one associated journal article. Our study began at the end of October 2018, which allowed at least six months to publish journal articles after each eligible DFRR was accepted.

Research projects funded under the broad PCORI announcement were eligible and included the following three priority areas: a) Addressing Disparities; b) Assessment of Prevention, Diagnosis, and Treatment Options; and c) Improving Healthcare Systems. Many of the eligible studies included intervention evaluations (e.g., randomized clinical trials). We excluded projects in the Communication and Dissemination Research priority area because they differ from other projects with respect to opportunities for spin; for example, they are not research reports and might not contain results regarding the effects of interventions. Similarly, research projects funded under the Improving Methods for Conducting PCORI announcement were excluded because these projects have different goals, such as comparing statistical methods, and might not contain the same types of spin as reports of comparative effectiveness research.

Because we sought to evaluate the potential effects of PCORI peer review on journal articles, we excluded projects with no associated journal article. Of the journal articles associated with an included project, we included those that described project results. We excluded protocols and other reports that did not describe results of the research described in the DFRR.

### Data collection

We obtained the eligible DFRRs, PCORI peer review feedback, and investigators’ responses to comments through Editorial Manager. Seven of the 64 (11%) eligible DFRRs were submitted by email before the editorial management system was in use, and we obtained these reports and PCORI peer review documents from available electronic records. We curated the list of articles associated with each DFRR from the publicly available PCORI webpage for each report. We did not search other scientific databases to identify additional articles associated with the DFRRs.

#### Draft final research reports (DFRRs)

For each eligible project, we recorded the date the DFRR was submitted; the date peer review feedback was sent to the principal investigator; the date the DFRR was accepted as completing peer review; and the accepted version number of the DFRR.

Rather than review each DFRR to assess the prevalence of spin, we recorded whether the PCORI peer review feedback included comments about spin. Two assessors independently read the feedback and completed a Google Form to record the types of spin that peer reviewers and editors identified in the DFRRs sent for peer review (i.e., in the first version of the DFRR). Peer reviewers and editors commented about many issues related to study rigor and reporting; we recorded those comments that explicitly raised issues related to “spin” as defined using the categories identified by Chiu and colleagues (Table 1) [7]. For each report, we coded the complete feedback sent to the investigators; we did not code individual reviewers’ or editors’ reports separately.

**Table 1:** Types and examples of spin evaluated in Patient Centered Outcomes Research Institute (PCORI) Draft Final Research Reports and publications

Category of spin	Strategy Used/Explanation, abstracted from Lazarus et al. and Chiu et al. [7, 8]	Examples of reviewers/editors’ comments
1. Reporting bias	ccNot reporting adverse events or lack of focus on harms (e.g., no warning on important safety issues), • Selective reporting of outcomes favoring the beneficial effect of the experimental treatment (e.g., statistically significant results for efficacy outcomes or statistically non-significant results for harm outcomes), • Misleading reporting of study design • Use of linguistic spin or “hype” (i.e., rhetorical manipulations to convince the readers of the beneficial effect of the treatment such as “excellent” results, “encouraging” outcomes, “a trend toward significance”), • No consideration of limitations	• Your report, particularly the Discussion, emphasizes “positive” results. • To report there was no main effect on a particular measure or to imply changes between different time points on a particular measure is misleading given the analyses conducted. • the authors should recalibrate the tone of the report, especially the Discussion and Conclusions sections, so that they are more balanced. • However, this reviewer still questions your terminology describing this outcome as “robust.”
2. Inappropriate interpretation	• Claiming a beneficial effect of the intervention despite statistically non-significant results, • Claiming an equivalent effect of the interventions for statistically non-significant results despite wide confidence interval • Claiming that the treatment is safe for statistically non-significant safety outcomes despite lack of power • Concluding a beneficial effect despite no comparison test performed • Interpretation of the results according to statistical significance (*p*-value) instead of clinical relevance	• The main concern here is whether the...score is statistically significant, and if it is, whether this small change is clinically meaningful • The effect, even when statistically significant, was quite modest clinically. • The results section would benefit from limiting statements to those that can be supported by the statistics conducted, avoiding speculation or general statements of effects, and minimizing the reporting of marginally significant findings. • Given the lack of a comparison group, evaluating effectiveness may not be possible. • There is too much self-selection bias to draw any meaningful conclusions from the results
3. Attribution of causality	• Claiming a causal effect between the intervention being assessed and the outcome of interest despite a non-randomized design	• The study design does not include a control group, which should be discussed as a limitation. • The discussion implies that the...result may be a reflection of confounding. If you believe this to be the case, I suggest mentioning it in the abstract so as not to impress on the reader that this is a causal finding.
4. Inappropriate extrapolation	• Extrapolation from the population, interventions or outcome actually assessed in the study to a larger population, different interventions or outcomes • Inadequate implications for clinical practice.	• More text on how the results, taken from a sample of convenience to academic medical centers in [elided], may not apply to other populations that face problems … • Furthermore, are these results, derived in a reasonably homogeneous population, generalizable to other underserved populations? Given the issues outlined in these comments, it would seem advisable to qualify some of your conclusions and to emphasize even more strongly that further data in a larger study are needed to say anything more conclusive. • The very low representation of people of color is troubling and should be discussed more at length in the results and discussion.

Examples of the reviewers/editors’ responses are from PCORI peer review feedback from the study sample

Assessors then used the DFRR as accepted by PCORI peer review and the investigators’ response letter to determine whether the investigators had addressed comments about spin either by modifying the report or by providing a justification (rebuttal) in their report or in their letter. Assessors did *not* judge whether they considered the accepted report to be free of spin; instead, assessors looked for evidence that the investigators made the specific changes recommended during peer review. We did not include a “partial” response category in our assessment. Instead, assessors coded that all changes were *not* made if the investigators addressed some but not all comments about spin *and* the investigators did not provide a justification for not addressing those comments. Assessors coded “changed the DFRR to address this issue” when they found evidence that investigators addressed all comments about spin by modifying the report. Assessors coded “provided a satisfactory response explaining why a change is not required” when the investigators explained or rebutted comments in a response letter and the assessor judged that the response included a reasonable scientific justification.

Next, the assessors compared their responses on the Google Form, discussed differences, reached agreement concerning the final rating, and entered a final response in a separate form for reconciled DFRR ratings. All final ratings used for analysis were agreed by both assessors. We did not calculate agreement for their preliminary ratings.

#### Journal articles

We assessed whether spin identified in the DFRRs was also present in related journal articles, and we compared journal articles accepted for publication before and after investigators received PCORI peer review feedback on corresponding DFRRs.

For each eligible journal article, two investigators checked the journal website and recorded the date of article acceptance or publication in a Google Form (Appendix). We checked these dates for agreement and resolved differences by re-checking the journal articles and journal websites.

If a journal article reported results for a study for which PCORI peer review had identified spin in the DFRR, then two investigators independently evaluated whether the spin identified in the DFRR was also present in the journal article (Appendix). Each reviewer entered their response into a Google Form (Appendix); assessors then compared ratings and entered a final response in a Google Form for reconciled journal article ratings. Because we used final reconciled ratings for analysis, we did not calculate agreement statistics.

### Outcomes and analyses

We combined data from the Google Forms for analysis by matching each DFRR with the articles associated with that DFRR. We calculated descriptive statistics for characteristics of the DFRRs and journal articles as proportions or as medians and interquartile ranges (IQRs) as appropriate. All analyses were completed using RStudio, version 1.2.1335.11 [11].

For the DFRRs, we calculated the proportions that were accepted by PCORI peer review after 1, 2, 3 or 4 revisions, and the proportions with articles accepted or published prior to receiving PCORI peer review feedback. We calculated the proportion of DFRRs with any spin identified, the proportion of DFRRS with each type of spin, and the proportion of DFRRs that addressed comments about spin in the accepted version. After beginning data extraction, we found some authors addressed comments about spin but did not change all relevant parts of their reports. For example, one reviewer suggested that the authors should address risk of confounding by indication in their abstract, but the authors added that limitation to their discussion in the main manuscript and not to the abstract. In this case, we judged that the report *had* addressed the comments about spin because the authors made some reasonable changes.

We also calculated the proportion of DFRRs that were associated with articles containing spin during the duration of our study. For articles that presented results, we calculated the proportion for which comments about the DFRRs were potentially applicable (i.e., articles describing parts of a project for which spin was identified during peer review of the DFRR); and for those we calculated the proportion of articles that included the spin identified in the DFRR.

For the journal articles that reported study results, we compared the date on which the journal article was accepted with the date on which PCORI peer review feedback was sent. We present stratified analyses of journal articles accepted prior to receiving PCORI peer review feedback, accepted after receiving PCORI peer review feedback, and for which we could not determine the timing of the journal articles compared with PCORI peer review feedback.

## Results

### PCORI research reports

**Fig. 1:**
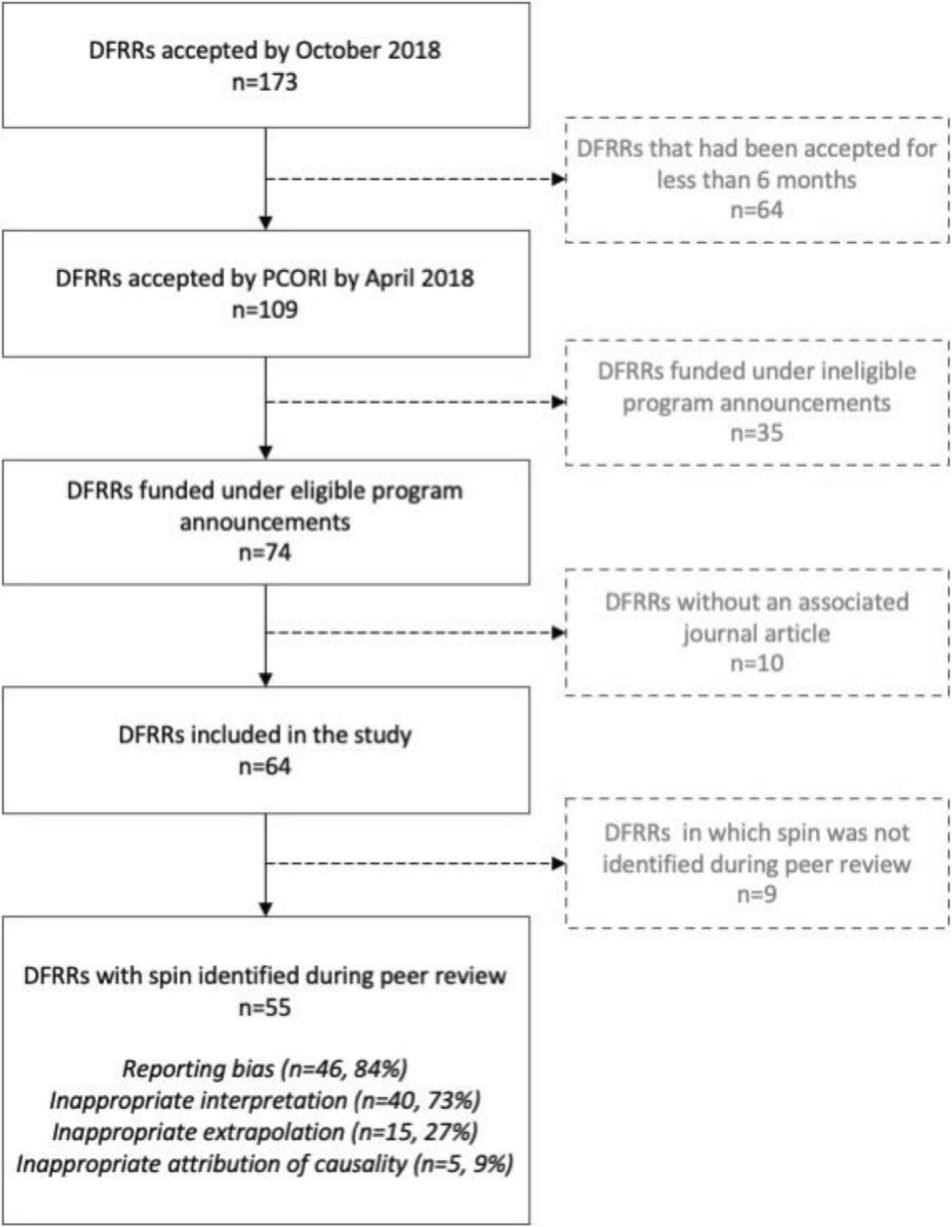
Flowchart for the inclusion of Draft Final Research Reports in this study

We included 64 eligible DFRRs with at least one associated journal article listed on the PCORI website (Fig. 1). We excluded 10 DFRRs with no associated journal articles, 35 reports that had completed review but were not related to an eligible priority area (e.g., methodologic studies), and 64 reports that completed review after the cutoff date for inclusion in our study (April 2018). All DFRRs were revised at least once before acceptance; most were accepted after the first or second revision: 20/64 (31%) and 31/64 (48%), respectively (Table 2).

**Table 2:** Characteristics of included PCORI studies and Draft Final Research Reports (DFRR)

*Characteristics*	*N (%)*
**Accepted version of DFRR (*N*** **= 64)**	
1st revision	20 (31%)
2nd revision	31 (48%)
3rd revision	11 (17%)
4th revision	2 (3%)
**Did the reviewers comment about spin in the DFRR? (N = 64)**	
Yes	55 (86%)
No	9 (14%)
**Did the project have one or more journal articles** ***accepted or published*** **before receiving PCORI peer review feedback? (N = 64)**^**a**^	
Yes	58 (90%)
No	5 (8%)
Can’t tell	1 (2%)
**In DFRRs with spin, which type of spin was identified? (*N*** **= 55)**	
Reporting bias	46 (84%)
Inappropriate interpretation	40 (73%)
Attribution of causality	5 (9%)
Inappropriate extrapolation of results	15 (27%)
**Among DFRRs with spin identified, were comments about spin addressed in last version of the DFRR? (N = 55)**	
Yes, the authors changed the DFRR to address this issue	38 (69%)
Yes, the authors provided a satisfactory response explaining why a change is not required^b^	9 (16%)
No	8 (15%)
Can’t tell	0 (0%)
**Of the DFRRs in which spin was identified, did at least one article include the spin identified in the DFRR? (N = 55)**	
Yes, one or more articles included spin	21 (38%)
No, none of the articles included spin	13 (24%)
Not applicable, none of the associated articles reported results related to comments about spin	21 (38%)
Can’t tell	0 (0%)

^**a**^We could not determine the exact date of acceptance for 88 articles, so we compared the date of publication with the date of the PCORI peer review feedback. Of those, 67 articles were published before sending PCORI peer review feedback and therefore must have been accepted before PCORI peer review feedback was sent. Two (2) articles were published within 28-days of sending PCORI peer review feedback and we assumed the articles were accepted before PCORI peer review feedback was sent. Of the remaining articles, 15 were published more than 98 days after PCORI peer review feedback was sent and we judged them likely to have been accepted for publication after the authors had received PCORI peer review feedback. We could not determine whether 4 articles published between 29 and 97 days of sending PCORI peer review feedback had been accepted before or after sending PCORI peer review feedback; three (3) of these articles were associated with DFRRs for which there was at least one article accepted prior to receiving PCORI peer review feedback

^b^In all cases for which assessors coded “provided a satisfactory response explaining why a change is not required,” the associate editors accepted the investigators’ reports without their previously recommended changes

#### Spin in DFRRs

We found that spin was identified frequently in submitted reports of PCORI-funded research, and there was less spin in subsequent draft reports following peer review.

Specifically, reviewers raised comments regarding spin in 55/64 (86%) of DFRRs. Of those more than one type of spin was detected in 38/55 (69%).

Of the DFRRs with spin, we identified comments about reporting bias in 46/55 (84%) of DFRRs, inappropriate interpretation in 40/55 (73%), inappropriate extrapolation of results in 15/55 (27%), and inappropriate attribution of causality in 5/55 (9%). In DFRRs, comments about reporting bias typically focused on the emphasis that investigators gave to outcomes with “statistically significant” results rather than the planned outcomes and analyses. Comments about inappropriate interpretation were typically related to reporting bias and the larger limitations of null hypothesis testing. Editors and reviewers identified fewer problems related to inappropriate extrapolation; mostly they suggested that authors should highlight limitations in their interpretations of results. Comments about inappropriate attribution of causality were relatively uncommon, and most were related to study design and the interpretation of associations. In addition to identifying critical comments about spin, we also found some positive comments about the lack of spin.

#### Spin in accepted PCORI research reports

Most authors addressed all peer review comments about spin by changing the text of their reports (38/55, 69%). Authors of several reports (9/55, 16%) justified their approaches or language in the investigators’ response letter, while other DFRRs were accepted without authors responding to comments about spin (8/55, 15%).

Some authors argued that their manuscripts did not contain spin. For example, one author responded to a comment about confounding and interpretation that because a variable mentioned by the reviewers would be in the causal path, it would be inappropriate to treat it as a potential confounder. In all cases in which we judged that investigators had responded to comments about spin by providing a scientific justification, the PCORI associate editors also accepted the research reports without further changes. The types of spin were similar in DFRRs that changed the report and DFRRs that rebutted comments about spin, including reporting bias (32/38, 84% versus 8/9, 89%), inappropriate interpretation (28/38, 74% versus 6/9, 67%), inappropriate extrapolation of results (9/38, 24% versus 2/9, 22%), and inappropriate attribution of causality (4/38, 11% versus 0/9, 0%).

Although we did not record systematically whether spin was introduced during peer review, we unexpectedly noticed two examples in which peer review encouraged spin. In one case, an associate editor encouraged the authors to focus on *p*-values and to interpret their results more positively, but the authors made minimal changes in their DFRR. In another case, the authors addressed comments about the interpretation of measures with clinically unimportant differences by removing all mentions of those results from their conclusions in the report.

### Associated journal articles

**Fig. 2:**
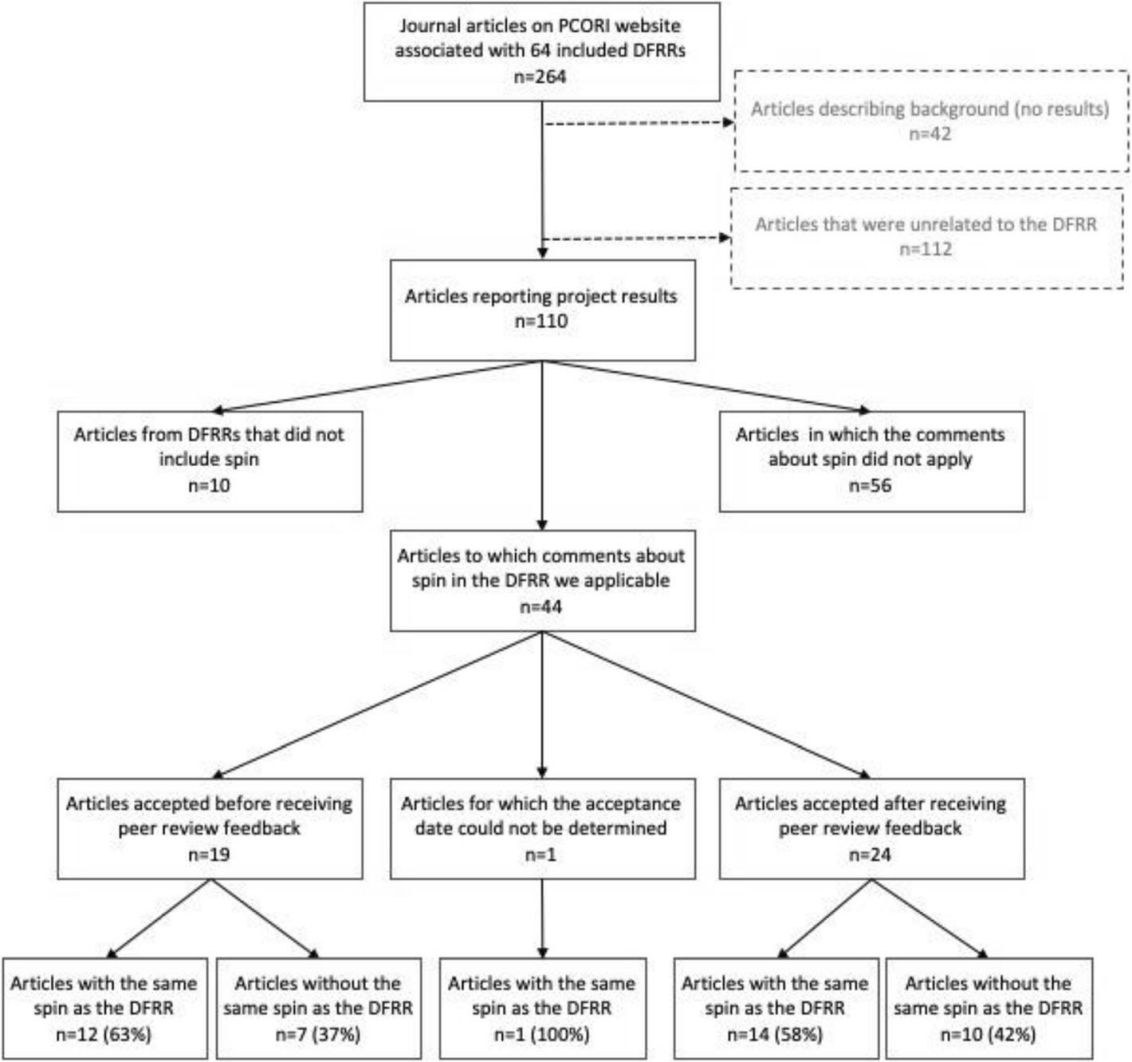
Flowchart for the inclusion of journal articles in this study

Using the PCORI website, we identified 264 journal articles associated with the included projects, with a median of 3 (IQR = 2 to 5) articles per project (Fig. 2). Of these, 110/264 (42%) reported study results (Fig. 2). We did not assess the prevalence of spin in 42/264 (16%) articles that did not report study results (e.g., protocols, reports of baseline characteristics). We also did not we assess the prevalence of spin in 112/264 (42%) articles on the PCORI website that did not appear to arise from the project described in the corresponding DFRR; these included commentaries, articles describing previous studies conducted by the investigators, and other communications that were not about the specific research funded by PCORI and described in the associated DFRR.

Of the articles describing project results, we determined that 63/110 (57%) were accepted before the corresponding PCORI peer review feedback was sent, and 44/110 (40%) were accepted after PCORI peer review feedback was sent. We could not determine whether 3/110 (3%) were accepted before or after the PCORI peer review feedback was sent (Fig. 2).

#### Spin in associated journal articles

We found no evidence that peer review of DFRRs was associated spin in journal articles. Indeed, we found that some journal articles contained spin even when authors removed spin from their PCORI research reports, including articles accepted after PCORI peer review.

We identified 44 journal articles with results for which comments about spin in the DFRR could have been applicable to the journal article. In the other articles with results, there were either no related comments about spin in the DFRR (10/110, 9%) or comments about spin made in the DFRR were not applicable to the parts of the project included in the journal article (56/110, 51%; (Fig. 2).

We found 27/44 (61%) articles contained spin that was also identified in the DFRR while 17/44 (39%) did not (Fig. 2). The proportion of articles with spin was similar for articles accepted before PCORI peer review (12/19; 63%) and accepted after PCORI peer review (14/24; 58%). We could not determine whether one article (1/44, 3%) was published before or after the DFRR was submitted.

For the 47 DFRRs that addressed all comments about spin in the first version of the DFRR (i.e., removing the spin or providing a rebuttal), we found 36 journal articles that could have included the same spin. Comparing those articles with the associated DFRRs, we found that 20/36 (56%) articles had the same spin as the first version of the DFRR and 16/36 (44%) did not have the same spin that was identified in the first version of the DFRR. Thus, the proportion of articles with spin was similar for those accepted before PCORI peer review (8/15; 53%) and accepted after PCORI peer review (11/20; 55%). As above, we could not determine whether one article was published before or after the DFRR was submitted (1/36, 3%).

## Discussion

Peer review frequently identified spin in reports submitted to PCORI, and spin identified by reviewers and editors was usually removed after review. Given that the PCORI Methodology Standards include expectations for reporting research, interventions to help investigators adhere to these standards before submitting their DFRRs might improve the quality of reports sent for peer review. We found no evidence that peer review of PCORI research reports was associated with removal of spin in associated journal articles.

Because the manner in which authors describe study results might influence how readers interpret study findings, spin in biomedical research might adversely affect the development of future studies, clinical practice, and health policies [6].

PCORI’s publication and peer review requirements are designed, in part, to reduce reporting bias; this study suggests that PCORI’s requirements might be having this intended effect. PCORI-funded investigators must publish a PCORI research report, which may reduce selective non-reporting of studies and results. For studies with null results for primary outcomes, we found peer review raised attention to primary outcomes rather than secondary outcomes with “significant” results. Peer review also encouraged authors to focus on between-group differences rather than within-group change, and to avoid interpretating null results in superiority trials as evidence of equivalence [12]. Some investigators over-generalized or over-interpreted exploratory analyses of heterogeneity, and peer review suggested greater consideration of study limitations. Inappropriate causal claims were relatively rare in DFRRs, perhaps because PCORI funds studies that are designed to allow strong causal inferences (e.g., many include randomized clinical trials). Peer review also suggested more appropriate causal language in several reports. Although we analyzed some of the first PCORI reports and peer review feedback, the peer review process is very similar today.

This study also provides some evidence that PCORI peer review is associated with better interpretation of results (i.e., reducing inappropriate interpretation, attribution, and extrapolation). In several cases of irreconcilable differences over important scientific matters, PCORI has published statements at the time of posting DFRRs that say PCORI disagrees with a study’s findings or interpretation, and provided their reasons behind those disagreements. Authors in those cases were invited to provide their rebuttals.

Although both journal articles and PCORI research reports are peer reviewed, differences in the peer review processes might explain observed differences in spin in reports about the same studies. The associate editors who review PCORI research reports are guided by the PCORI Methodology Standards, and PCORI will publish all research reports without respect to the novelty of the findings. The proportion of journal articles with spin in our study was comparable to previous studies; for example, Boutron and colleagues found spin in 58% of the Conclusions in the abstracts for trials with nonsignificant primary outcomes [6]. Further research is needed to determine whether these features of PCORI peer review could reduce spin in journal articles.

PCORI associate editors and peer reviewers made many comments on DFRRs, which included comments unrelated to spin (e.g., handling of missing data, absence of confidence intervals, incomplete participant flow diagrams). While most investigators changed their research reports in response to comments about spin, our finding that not all investigators resolved comments about spin could be evidence that investigators disagree with what constitutes “spin,” or that comments about spin were perceived by the investigators (and by the editors who handled those research reports) as relatively less important than other comments. Evidence that investigators sometimes responded with justifications rather than changes suggests that investigators and editors sometimes disagree about what constitutes “spin.” We did not independently evaluate DFRRs and peer reviewers’ comments about spin, and we are not aware of a reference standard that could be used to evaluate the agreement of different readers for detecting spin. A previous study found that peer reviewers identified spin in most abstracts for non-randomized intervention studies, yet reviewers did not comment about all instances of spin [8, 9].

Our finding that spin was commonly identified in PCORI research reports during peer review is consistent with studies showing that selective non-reporting and spin originate with authors rather than with journal editors or journalists [13–16]. In addition to external reviews, PCORI feedback to investigators included comments from Associate Editors who were trained to identify and address to address problems that include spin. Comparing PCORI research reports and journal articles, we also found that different reports of the same study may contain different spin. Our results are consistent with previous studies showing that multiple reports about the same study sometimes contain different information about study design, risk of bias, outcomes, and results [17, 18]. This study also suggests that differences in peer review might contribute to differences in spin when comparing PCORI research reports and journal articles. We do not know whether corresponding authors of PCORI research reports shared comments with their co-investigators, and we did not compare authors listed in each PCORI research report and journal article, so we cannot know whether comments about the research reports could have influenced all authors of the journal articles.

Although our study did not aim to compare text across sources per se, we found that many PCORI research reports and journal articles contained similar text. Consequently, it might be difficult for readers to see how a PCORI report differs from journal articles about the same study. Future studies could explore whether PCORI authors reference their PCORI reports in other publications, and whether stakeholders use PCORI research reports when journal articles are available for the same study.

### Limitations

Our study had several limitations. First, we evaluated comments about spin related to only the first version of each DFRR. Many DFRRs were submitted multiple times, and comments about the first version sometimes focused on issues such as clarity, completeness, and analytic methods. It is possible that associate editors commented about spin on subsequent drafts and that we underestimated the proportion of PCORI research reports with comments about spin. For example, we cannot exclude the possibility that all reports eventually received some comments about spin. It is also possible that editors and peer reviewers might have considered some of the investigators’ justifications to be inadequate; because we did not look at acceptance letters (which do not tend to include many additional comments to investigators), we do not have any direct evidence about the editors’ beliefs about the investigators’ responsiveness to their comments. In addition, our analysis focused on the peer review process that precedes PCORI’s final review. We did not consider comments provided by the PCORI Director of Peer Review, who might have also identified spin. Although all studies and articles were rated by at least one person not involved in PCORI peer review, the first author is an Associate Editor for PCORI peer review and rated some of the included DFRRs and journal articles. Despite these limitations, our conclusions would be the same if the true proportion of DFRRs with spin were greater than 86% and if the true proportion of PCORI research reports that addressed all comments about spin were slightly lower than 85%. Moreover, our conclusions would be unlikely to change meaningfully if comments from the PCORI Director of Peer Review led to additional changes in some PCORI reports and associated journal articles.

Another limitation was that we evaluated only journal articles identified by PCORI. We did not search for additional journal articles related to the PCORI research reports and we might have missed articles that would have been included had they been identified. However, we have no reason to believe that other articles published by the time of our study would differ meaningfully from the articles that we included. It is possible that articles published after the cutoff for our study might have addressed comments about spin that authors received during peer review of their DFRRs or from the PCORI Director of Peer Review.

Lastly, we used the date that each journal article was accepted—rather than the date each journal article was submitted—because journals more commonly report the date of acceptance than the date of submission. Although using the date of acceptance might overestimate the number of articles that could have been affected by PCORI peer review, using the date of submission might have underestimated the number of articles that could have been affected by PCORI peer review. Even if more complete information had been available for our study, we doubt it would change our conclusion that PCORI peer review was not associated with spin in journal articles about the same studies.

## Conclusions

PCORI research reports are comprehensive accounts of PCORI funded studies, and our analysis shows that these reports included less spin after peer review. Funders could explore interventions aimed at reducing spin in published articles of studies they support.

## Supplementary Information

**Additional file 1:.** Appendix

## Data Availability

Research materials are available on the Open Science Framework (https://osf.io/fh7cz/).

## Abbreviations

**PCORI**: Patient-Centered Outcomes Research Institute
**DOI**: Digital Object Identifier
**DFRR**: Draft Final Research Report
