# Supplementary material for: Peer review reduces spin in PCORI research reports
Source: Res Integr Peer Rev. 2021 Dec 1;6:16. doi: 10.1186/s41073-021-00119-1 (PMC8638354; doi:10.1186/s41073-021-00119-1)
Supplement: Supplementary file 1 — Additional file 1:. Appendix [file 41073_2021_119_MOESM1_ESM.zip › Appendix_02_Article_publication_information_form.pdf]

Article Publication Date Collection

This form is to collect the date of publication for articles that do not have either received or acceptance date.

* Required

Extractor

Two letter initial

Your answer

EM Manuscript Number *

Choose

Article number (which journal article from excel file)

- 1
- 2
- 3
- 4
- 5
- 6
- 7
- 8
- 9
- 10
- 11
- 12
- 13
- 14
- 15
- 16
- 17
- 18
- 19
- 20
- Option 21

DOI

Copy and paste the DOI for each article (after the http://doi.org/)

Your answer

Date of Publication

MM DD YYYY

/ /

From what type of publication does the date come?

- ◯ Electronic publication date
- ◯ Print publication date
- ◯ Can't tell

Submit

Never submit passwords through Google Forms.

This form was created inside of Indiana University. Report Abuse

Google Forms
