# Supplementary material for: Peer review reduces spin in PCORI research reports
Source: Res Integr Peer Rev. 2021 Dec 1;6:16. doi: 10.1186/s41073-021-00119-1 (PMC8638354; doi:10.1186/s41073-021-00119-1)
Supplement: Supplementary file 1 — Additional file 1:. Appendix [file 41073_2021_119_MOESM1_ESM.zip › Appendix_03_Article_Information_Form.pdf]

# PCORI Articles Form

Items needed for completion of this form: "Excel file for PCORI studies and Journal Articles" and journal articles

## B1. Extractor

Select the extractor's initials

- ○ AC
- ○ JG
- ○ EMW

## B2. EM Manuscript Number

Locate the EM Manuscript number from the "Excel File for PCORI studies and Journal Articles_20181130"

Choose ▾

## B3. Article number (which journal article from excel file)

Choose ▾

## B4. DOI

Copy and paste the DOI for each article (after the (after the http://doi.org/)

Your answer

## B6. Date received

Enter the date "Article was received" located on the journal article

MM DD YYYY

/ /

## B7. Date accepted

Enter the date "Article was accepted" located on the journal article

MM DD YYYY

/ /

**Submit**

Never submit passwords through Google Forms.

This form was created inside of Indiana University. Report Abuse

Google Forms
