# Supplementary material for: Peer review reduces spin in PCORI research reports
Source: Res Integr Peer Rev. 2021 Dec 1;6:16. doi: 10.1186/s41073-021-00119-1 (PMC8638354; doi:10.1186/s41073-021-00119-1)
Supplement: Supplementary file 1 — Additional file 1:. Appendix [file 41073_2021_119_MOESM1_ESM.zip › Appendix_04_Article_independent_rating_form.pdf]

Articles Form for SPIN Review

Items needed for completion of this form: Excel File for PCORI studies, journal articles, Synthesis Letter

B1. Extractor

Select the extractor's initials

- ◯ AC
- ◯ JG
- ◯ EMW
- ◯ MP

B2. EM Manuscript Number

Locate the EM Manuscript number from the "Excel File for PCORI studies and Journal Articles_20181130"

Choose ▾

B3. Article number (which journal article from excel file)

Choose ▾

B4. DOI

Copy and paste the DOI for each article (after the http://doi.org/)

Your answer

B5. Does this journal article describe project results? If NO, enter notes in B7 and/or submit form.

- [ ] Yes
- [ ] No- describes study background
- [ ] No- article is unrelated to study

B6. Does the journal article include the problems related to spin identified during OHSU peer review?

Review the article's abstract, results, and discussion for spin identified in question A4 from the New Spin in the DFRR Form

- ◯ Yes
- ◯ No
- ◯ Not applicable because the comments about spin do not apply to this journal article
- ◯ Not applicable because spin was not identified during the OHSU peer review

B7. Notes about spin in this study

Your answer

Submit

Never submit passwords through Google Forms.

This form was created inside of Indiana University. Report Abuse

Google Forms
