# Supplementary material for: Peer review reduces spin in PCORI research reports
Source: Res Integr Peer Rev. 2021 Dec 1;6:16. doi: 10.1186/s41073-021-00119-1 (PMC8638354; doi:10.1186/s41073-021-00119-1)
Supplement: Supplementary file 1 — Additional file 1:. Appendix [file 41073_2021_119_MOESM1_ESM.zip › Appendix_05_DFRR_reconciled_rating_form.pdf]

Reconciliation of Spin in the DFRR

Items needed for completion of this form: Instructions Manual, Synthesis Letter, and Final Research Report (FRR)

A2. EM Manuscript Number

Locate the EM Manuscript Number from the JHU Master Peer Review Data Sheet

Choose

A3. Did the reviewers/editors comment about spin?

From the Synthesis Letter evaluate for any comments about spin. If response is NO or Can't tell, skip to A6

- Yes
- No (skip to question A6)
- Can't tell (skip to question A6)

A5. Types of spin identified

Identify the type(s) of spin reported utilizing examples from SPIN Table from the Instructions Manual (select all that apply)

- [ ] Reporting bias
- [ ] Inappropriate interpretation
- [ ] Attribution of causality
- [ ] Inappropriate extrapolation
- [ ] Can't tell

SPIN categories

Category of spin	Strategy Used/Explanation, abstracted from Lazarus et al.[3]
1. Reporting bias	• Not reporting adverse events or lack of focus on harms (e.g., no warning on important safety issues),<br>• Selective reporting of outcomes favoring the beneficial effect of the experimental treatment (e.g., statistically significant results for efficacy outcomes or statistically non-significant results for harm outcomes),<br>• Misleading reporting of study design<br>• Use of linguistic spin or "hype" (i.e., rhetorical manipulations to convince the readers of the beneficial effect of the treatment such as "excellent" results, "encouraging" outcomes, "a trend toward significance"),<br>• No consideration of limitations
2. Inappropriate interpretation	• Claiming a beneficial effect of the intervention despite statistically non-significant results,<br>• Claiming an equivalent effect of the interventions for statistically non-significant results despite wide confidence interval<br>• Claiming that the treatment is safe for statistically non-significant safety outcomes despite lack of power<br>• Concluding a beneficial effect despite no comparison test performed<br>• Interpretation of the results according to statistical significance (p-value) instead of clinical relevance
3. Attribution of causality	• Claiming a causal effect between the intervention being assessed and the outcome of interest despite a non-randomized design
4. Inappropriate extrapolation	• Extrapolation from the population, interventions or outcome actually assessed in the study to a larger population, different interventions or outcomes<br>• Inadequate implications for clinical practice.

A6. Does the last revision of the DFRR address peer review comments about spin/limitation ?

Reviewing the FRR and Question A4, answer the question below

- Yes - The authors changed the DFRR to address this issue
- Yes - The authors provided a satisfactory response explaining why a change is not required.
- No
- Can't tell

A7. Notes about Spin in this study

Describe any information that could not be extracted because of how data were presented in the report.

Your answer

Submit

Never submit passwords through Google Forms.

This form was created inside of Indiana University. Report Abuse

Google Forms
